# Nurse supervised combined refeeding and home parenteral nutrition in traumatic intestinal failure: A case series

**DOI:** 10.1016/j.ijscr.2019.07.049

**Published:** 2019-07-23

**Authors:** Adeodatus Yuda Handaya, Victor Agastya Pramudya Werdana, Aditya Rifqi Fauzi

**Affiliations:** a Digestive Surgery Division, Department of Surgery, Faculty of Medicine, Universitas Gadjah Mada/Dr. Sardjito Hospital, Jl. Kesehatan No. 1, Yogyakarta 55281, Indonesia; b Faculty of Medicine, Universitas Gadjah Mada/Dr. Sardjito Hospital, Yogyakarta 55281, Indonesia

**Keywords:** IF, intestinal failure, HPN, home parenteral nutrition, CVC, central venous catheter, SGA, subjective global assessment, Nurse supervised, Home parenteral nutrition, High output jejunostomy, Post-traumatic intestinal failure, Reanastomosis

## Abstract

**Background:**

Intestinal failure is a decrease in intestinal function under the minimum absorption requirements of macronutrients, water, and electrolytes. Hartman's procedure with jejunostomy is used as a surgical procedure to prevent further damage in cases of abdominal trauma. Providing parenteral nutrition at home is needed to prevent nutritional deficiencies and prolonged length of stay.

**Presentation of case:**

We reported two cases, involving two men aged 25 and 14 years old who had peritonitis due to abdominal trauma and received laparotomy. Both patients had Hartman’s procedures and jejunostomy less than 60 cm from the Treitz ligament. Both patients were diagnosed as bowel failure with an SGA C score. Supervised home parenteral nutrition was done by refeeding jejunostomy at the distal stoma. After supervision of parenteral nutrition, the SGA score increased from C to B. Three months later the patients underwent jejunal reanastomosis. Patients went home one week later without complications. At postoperative follow-up at one month and one year, both patients did not experience any complications.

**Discussion:**

HPN is now a method used to provide nutritional support for patients with IF. This helps patients to meet their nutritional needs, also preventing psychosocial disorders and reduction of their quality of life.

**Conclusions:**

Based on these two cases, nurse supervised combined refeeding and home parenteral nutrition showed good results with an increase in nutritional status of SGA C to SGA B. Supervision of home parenteral nutrition can be considered as adjunctive therapy in patients with high intestinal failure before undergoing reanastomosis.

## Highlights

• Combined refeeding and home parenteral nutrition in traumatic intestinal failure.
• Peritonitis due to abdominal trauma.
• Received Hartman’s procedure and jejunostomy.
• Parenteral nutrition at home.
• Prevent prolonged length of stay at hospital.

## 1 Background

Intestinal failure (IF) is defined as a decrease in the intestinal function below the minimum required for the absorption of macronutrients, water, and electrolytes so that intravenous supplements (IVS) are needed to maintain body health and growth [1,2]. Based on the expected onset, metabolic and outcome criteria, IF is classified into: a) type I, is acute, short-term, and often self-limiting; b) type II, acute but prolonged, often occurs in patients who are metabolically unstable, requiring complex multidisciplinary care and intravenous supplementation for several weeks or months; and c) type III, chronic, often occurring in patients who are metabolically stable, usually requiring months or even years of intravenous supplementation, can be recurrent or irreversible [1,3].

Home parenteral nutrition (HPN) is a method used to provide nutritional support for patients with intestinal failure [4]. HPN has become the standard of care for intestinal failure type III for decades. This regimen can be administered in the long term through percutaneous intravenous catheters, special pumps and by trained patients or specialized nursing staff [5]. This research work has been reported in line with the PROCESS criteria [6].

## 2 Case presentation

**Fig. 1:**
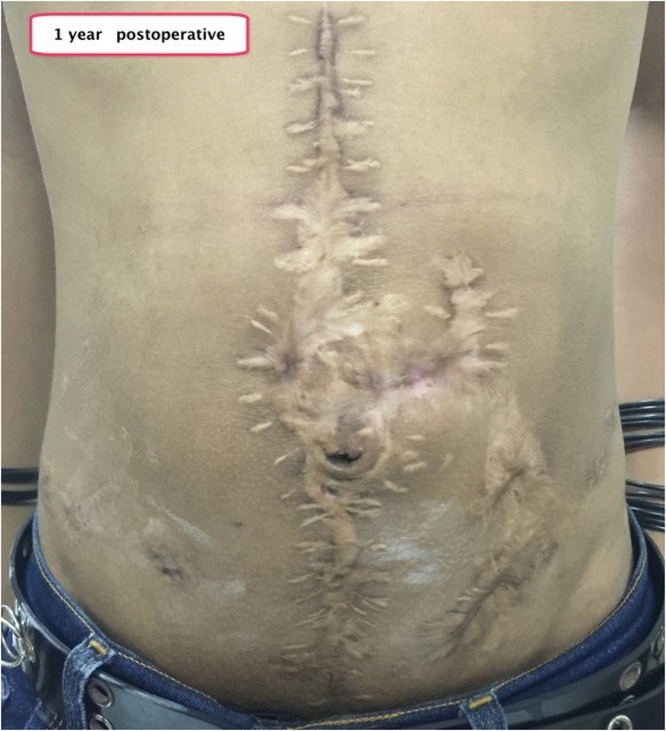
1-Year postoperative.

**Fig. 2:**
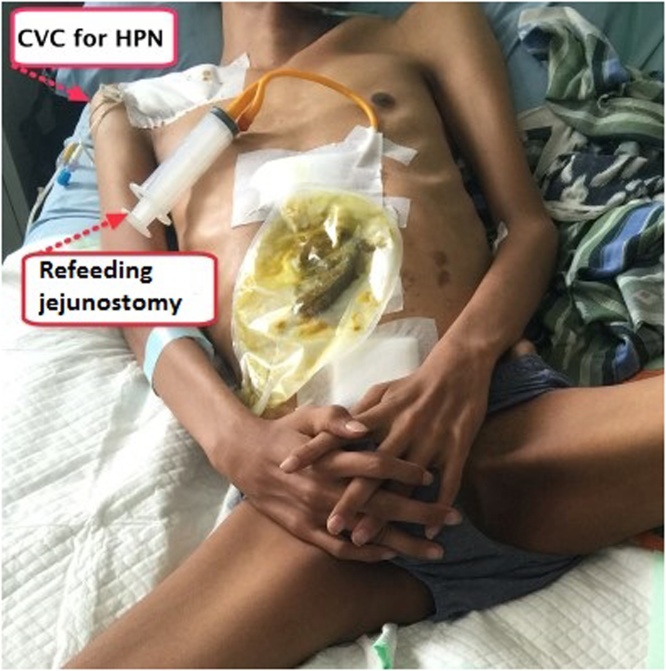
CVC for HPN and refeeding jejunostomy.

We reported two patients, involving young men aged 25 and 14 years who had peritonitis due to blunt abdominal trauma. Patients received laparotomy damage control surgery. Both patients underwent a Hartman procedure and jejunostomy approximately 60 cm from the Treitz ligament. The remnant of small bowel length of both patients are 40 cm and 60 cm, respectively. All patients produced high output jejunostomy (1000–1500 cc) per day and were diagnosed as an intestinal failure with nutritional status subjective global assessment (SGA) C (severe malnutrition). Both patients received home parenteral nutrition (partial parenteral nutrition with oral intake maintained) and supervised jejunostomy refeeding output to distal stoma by trained nurses for three months before finally planned for re-anastomosis. After three months, the nutritional status rose to SGA B. Then both patients underwent jejunal re-anastomosis. The patients were treated for one week in a post-operative hospital. No postoperative complications were seen. At the first and one-year postoperative evaluation, we found no complications (Fig. 1, Fig. 2).

## 3 Discussion

Intestinal failure is a severe complication from conditions such as inflammatory bowel disease, mesenteric ischemia, and extensive bowel resection caused by these diseases. Fluid and electrolyte imbalances, and poor nutritional status manifest as chronic diarrhea or increased ostomy output [9]. IF can occur due to obstruction, dysmotility, surgical resection, congenital defects or diseases related to loss of absorption. IF is characterized by the inability to maintain protein-energy, fluids, the balance of electrolytes and micronutrients [5].

HPN is now a method used to provide nutritional support for patients with intestinal failure [4]. HPN allows patients with IF to stay outside the hospital and meet their nutritional needs. The survival of patients depends mainly on the underlying disease. IF, including HPN-related complications, and associated social restrictions, often lead to psychosocial disorders and reduce the quality of life of the patients [7]. Use of central venous catheter (CVC) also requires special care [8].

The patient's medical needs determine short-term or long-term parenteral nutritional needs. Patients who need short-term parenteral nutrition (2–6 weeks) are those whose bowel function has not returned to normal postoperatively and patients who are severely malnourished before surgery while patients who need it in the long term (months to years, even lifetime) are patients with gastrointestinal and short bowel dysmotility syndrome due to extensive intestinal resection [9].

Subcutaneous fluid administration can be considered in patients with a simple fluid, sodium and/or magnesium deficit where parenteral fluid needs are less than 1 L/day. Placement of the subcutaneous cannula every night (6–10 h) usually allows the administration of at least 500 ml of fluid. In trauma settings, subcutaneous cannula can be used for resuscitation because it is more patent than the peripheral access. Patients should be warned that swelling can occur, but can diminish in the next few hours [10].

After surgery, the patient's stoma usually has high-output. In patients with high-output jejunostomy, fluid correction and electrolyte balance cannot be achieved with enteral feeding without accessible mucosal fistulas or refeeding tubes [11,12].

In addition to jejunostomy, another procedure that is often used to make venting ostomy is an ileostomy and also a gastrostomy. In severe cases, the laparotomy may only be limited through the upper left quadrant incision and create a distal loop of jejunostomy as distal as possible which is still safe to make, serving only to facilitate wound care [13].

## 4 Conclusions

In conclusion, nurse supervised home parenteral nutrition showed good results on two cases, with the outcome of improving the nutritional status of the patients from SGA C to SGA B. Patients, also did not experience complications of leakage or postoperative infection, while the digestive function also recovered. This method can be considered as adjunctive therapy for high-output traumatic intestinal failure before undergoing re-anastomosis.

## Sources of funding

The authors declare that this study had no funding resource

## Ethical approval

The informed consent form was declared that patient data or samples will be used for educational or research purposes. Our institutional review board also do not provide an ethical approval in the form of case report.

## Consent

Written informed consent was obtained from the patient for publication of this case report and accompanying images. A copy of the written consent is available for review by the Editor-in-Chief of this journal on request. Written informed consent was also obtained from the parent of the minor subject for this study.

## Author’s contribution

Adeodatus Yuda Handaya conceived the study. Aditya Rifqi Fauzi and Victor Agastya Pramudya Werdana drafted the manuscript and critically revised the manuscript for important intellectual content. Adeodatus Yuda Handaya, Aditya Rifqi Fauzi, and Victor Agastya Pramudya Werdana facilitated all project-related tasks.

## Registration of research studies

researchregistry4935.

## Guarantor

Adeodatus Yuda Handaya.

## Provenance and peer review

Not commissioned, externally peer-reviewed.

## CRediT authorship contribution statement

**Adeodatus Yuda Handaya:** Conceptualization, Methodology, Resources, Writing - original draft. **Victor Agastya Pramudya Werdana:** Writing - review & editing, Resources, Validation. **Aditya Rifqi Fauzi:** Writing - review & editing, Resources, Validation.

## Declaration of Competing Interest

No potential conflict of interest relevant to this article was reported.
